# Objectively measured adherence may affect side effects of mandibular advancement therapy in subjects with obstructive sleep apnea

**DOI:** 10.1007/s11325-023-02959-1

**Published:** 2023-12-19

**Authors:** Riitta Pahkala

**Affiliations:** Department of Oral and Maxillofacial Diseases, Kuopio University Hospital, P. O. Box 100, 70029 Kuopio, Finland

**Keywords:** Oral appliance, Obstructive sleep apnea, Temporomandibular disorders, Side effects, Dental changes, Adherence to treatment

## Abstract

**Purpose:**

The purpose of this study was to determine if objectively measured adherence to oral appliance (OA) treatment may affect dental changes and temporomandibular disorders (TMD) in patients with obstructive sleep apnea (OSA).

**Methods:**

The original study group consisted of adults with OSA who were referred for OA therapy. Eight indicators of subjective side effects of using OA (temporomandibular joint (TMJ) and muscle pain, pain in teeth, jaw stiffness in the morning, clicking, dry mouth, hypersalivation, gingival irritation) were evaluated by a questionnaire. Three occlusal indicators (overjet, overbite, molar occlusion) and clinical TMD signs (TMJ pain, muscle pain, clicking, jaw deviation on opening) were evaluated at baseline and at the 3-, 6-, and 12-month follow-up. In addition, objective adherence monitoring for OA was registered. Statistical analyses included the chi-square test, Fisher’s exact test, paired sample *t*-test, and linear regression analyses.

**Results:**

A total of 58 adults with OSA were referred for OA therapy. Mean (SD) age was 50.7 (11.7) and mean apnea-hypopnea index (AHI) was 19.5 (10.0). At 1-year follow-up, the study group consisted of 28 men and 12 women. Overjet but not overbite reduced significantly after 1-year OA therapy. The average nightly wear of OA was related to overjet and overbite reduction, and to TMD signs. Hypersalivation, dry mouth, and tooth discomfort were the most common subjective side effects of OA therapy.

**Conclusion:**

There was a time-dependent relationship with the nightly wear of OA and reduction in overjet and overbite, and clinical TMD signs. With 60% of mandibular advancement, dental changes and TM-disorders were considered mild/minor in the 1‐year study period.

## Introduction

Oral appliances (OA) for obstructive sleep apnea (OSA) are effective alternatives to continuous positive airway pressure (CPAP) therapy, especially for those who do not tolerate nor want to use CPAP [1, 2]. Considering the chronic nature of OSA, the use of an oral appliance is likely to be a lifelong treatment. Earlier studies have found that OAs have both short-term and long-term side effects such as dental/occlusal changes and signs and symptoms of temporomandibular disorders (TMD) [1–5]. It is suggested that the more one wears an OA, the more detrimental are the side-effects [6, 7]. Dental changes are visible early during the first year of OA use [8], and they are usually progressive with ongoing OA therapy [9]. Although dental changes do not usually disturb the subject, they are irreversible and thus may be clinically relevant [7]. Earlier review articles have found the following orofacial and dental changes with long-term OA use: retroclination of maxillary incisors, proclination of mandibular incisors, reduction in overjet (OJ) and overbite (OB), mandibular downward rotation, and increase in the lower facial height [10, 11]. Short-term temporomandibular disorders (TMD) during acclimatization to OA therapy are common, but they are usually temporary and disappear during the first few months of treatment [2, 4]. However, TM disorders may also be unchanged in long-term follow-up [12]. Altogether, moderate/severe side effects have a profound effect on adherence to OA therapy [13], and in addition to ineffectiveness of OA in decreasing OSA signs and symptoms, they are the major reasons for discontinuation of OA therapy [14]. The highest percentage of interruptions in OA use is likely to occur during the first year of appliance use [14].

The risk of side effects increases by augmentating the degree of protrusion of the mandible [15]. Therefore a moderate mandibular advancement is highly recommended. Regarding treatment outcomes among patients with OSA, our earlier results showed that more than half of patients attained a complete response (AHI < 5 events/h) to OA therapy. The prevalence of snoring also decreased significantly, and the upper airway space improved remarkably with 60% of mandibular advancement using the oral device [16]. The aim of this study was to determine if there were less subjective symptoms and TMD signs, as well as minor dental side effects with this treatment protocol. Another focus of this study was to determine if the objectively measured adherence to oral appliance therapy was related to the risk of dental and temporomandibular side effects.

## Subjects and methods

### Subjects

This prospective study included consecutive patients diagnosed to have OSA by ambulatory polygraphic recording and who were referred for oral appliance therapy to the Oral and Maxillofacial Department, Kuopio University Hospital. Subjects were enrolled if they were 18 years or older, their AHI was at least ten events/h, their body mass index (BMI) was less than 35 kg/m^2^, and they had at least five teeth/jaw. The subjects were examined at baseline and 3, 6, and 12 months after OA therapy. The Research Ethics Committee of the Hospital District of Northern Savo in Kuopio, Finland, approved the study protocol on February 7, 2017 (80/2017). All patients supplied a written informed consent before participating in the study. The original study flowchart is elsewhere [16].

### Methods

Nocturnal single-night ambulatory polygraphic recordings were conducted at home to diagnose OSA prior to this study and after 3 months with the oral appliance (OA) in situ. Apneas and hypopneas were automatically scored (Remlogic, version 3.2, and Somnologica, version 3.2 software, Embla Co., Broomfield, CO, USA) and manually verified and edited. Trained physicians evaluated the recordings. The present part of the study included the following data:

Descriptive data: age (years), gender, follow-up time of OA therapy (3, 6, and 12 months), mandibular protrusion with OA (mm), average nightly use of OA (hours) and regular use of OA (use of OA at least 4 h/night on at least 70% of all nights).

Clinical examination: Occlusal findings were assessed intraorally according to the modified method of Björk [17] including molar occlusion (anteroposterior upper and lower first molar relationship), overjet (OJ, the horizontal distance between the upper and lower front teeth), and overbite (OB, the vertical overlap between the upper and lower front teeth). Also, the number of missing teeth was recorded. Signs and symptoms of TMD and jaw movements were evaluated by the modified method of Helkimo [18] including palpatory tenderness of the masticatory muscles and the temporomandibular joints (TMJs), clicking, jaw deviation on maximal opening, and mandibular mobility (Table 1). The original study protocol did not include cephalometric measurements. The trained orthodontist (RP) examined all the patients.

**Table 1:** Anamnestic and clinical dysfunction index according to Helkimo

Anamnestic dysfunction index, Ai	Clinical dysfunction index, Di
Ai0 denotes subjects who are free from any symptoms of dysfunction in the masticatory system Ai I denotes mild symptoms such as temporomandibular joint sounds (clicking and crepitation), feeling of stiffness or fatigue of the jaws Ai II denotes severe symptoms of dysfunction. At least one of the following symptoms were reported: difficulty in opening the mouth wide, locking, luxation, pain on movement, facial and jaw pain	Di 0 denotes absence of the clinical symptoms Di I denotes mild symptoms of dysfunction. 1–4 of the following symptoms were recorded: deviations of the mandible in opening and/or closing movement > 2 mm from the straight line, TMJ sounds (clicking and crepitation), tenderness to palpation of the masticatory musculature in 1–3 palpation sites, tenderness to palpation laterally over the TMJ, pain on one movement of the mandible, slightly impaired mobility of the mandible Di II denotes moderate symptoms of dysfunction and at least one severe symptom. The severe symptom may be any of the following: locking/luxation of TMJ, tenderness of palpation at least 4 sites of the masticatory musculature, tenderness to palpation posteriorly of the TMJ, pain at least in two movements of the mandible, maximal mouth opening < 30 mm, one or more horizontal movement < 4 mm Di III denotes 2–5 of the severe symptoms possibly combined with any of the mild symptoms

*TMJ* temporomandibular joint

Questionnaire of the treatment-related side effects (dichotomous scale): Pain in TMJ and in masticatory muscles, clicking, and jaw stiffness in the morning were assessed by the modified method of Helkimo [18]. In addition, patients were inquired (yes/no) about discomfort in teeth, gingival irritation, hypersalivation, and dry mouth (Table 2). The patients got the questionnaires at each follow-up visit, and they were asked to complete the questionnaires and return them by mail.

**Table 2:** A questionnaire for subjective side effects during OA therapy

1) Do you have pain in teeth?	Yes	No
2) Do you have pain in the region of TMJs?	Yes	No
3) Do you have sound (clicking or crepitation) in the region of TMJs?	Yes	No
4) Do you have pain in masticatory muscles?	Yes	No
5) Do you feel stiffness during awakening or movement of the jaw?	Yes	No
6) Do you have difficulty while opening the mouth?	Yes	No
7) Do you have the feeling of occlusal changes?	Yes	No
8) Do you have gingival irritation?	Yes	No
9) Do you have hypersalivation?	Yes	No
10) Do you have dry mouth?	Yes	No

Mandibular advancement device therapy: Patients used SomnoDent Flex (SomnoMed Ltd, Sydney, Australia) custom-made acrylic duo block titratable oral device with a temperature-sensitive micro-recorder (DentiTrack^R^, Braebon, Ontario, Canada) to measure the objective adherence during OA therapy at 3, 6, and 12 months of follow-up. The recorder registers the wearing time of the device, and by using the base station, the data is uploaded into the Health Insurance Portability and Accountability Act of 1996 (HIPAA)-secure cloud. For each patient, the average OA wear in hours per night and in percentage of nights per week was recorded and was referred to as the objective mean wearing time. In the statistical analyses, frequent users were the ones whose objective wear of OA was at least 4 h/night over 70% of all the nights. In SomnoDent Flex, the adjustable interlocking acrylic buccal extensions connect the upper and lower splints. In this study, advancement of the mandible with OAs was determined as 60% of the voluntary maximal retrusion to maximal protrusion of the mandible. Advancement was assessed by SOMGauge bite registration device. In SomnoDent Flex, there is a screw mechanism to advance the jaw if any titration of the device is needed. No routine mandibular jaw exercises to cope with side effects were given to the patients.

### Statistical analysis

For statistical analyses, the IBM SPSS statistics, version 22.0 (IBM Corp., Armonk, NY, USA) was used. The chi-square test was used to analyze the differences in categorical variables between males and females. Fisher’s exact test was used when the numbers of subjects in some cells were small. The differences in continuous variables were analyzed using paired samples *t*-test for normally distributed variables. Multivariate linear regression analysis was used to investigate the associations of age, gender, average nightly wear of OA, and mandibular protrusion with OA with a reduction of OJ and OB during 1 year of OA treatment. In addition, multivariate linear regression models were used to evaluate the associations between the sum of temporomandibular dysfunction signs (muscle tenderness, clicking, TMJ tenderness, deviation on opening) and those independent variables at the 3-month, 6-month, and 12-month follow-up. The independent variables were added simultaneously in regression analyses, and their choices except for age and gender were based on the outcomes in bivariate analyses. Associations with *p* values of < 0.05 were statistically significant.

## Results

There were 28 men (mean age [SD] 49.2 [12.6]) and 12 women (mean age [SD] 58.3 [5.4]) who attended the 12-month follow-up. The results showed that during 1-year study interval OJ and OB reduced 0.36 mm and 0.25 mm, respectively (Table 3). Only in OJ, the reduction was statistically significant. Concerning molar occlusion, only four men and four women had occlusal changes after 1‐year of OA treatment (Table 4). The mean number of missing teeth per patient was 2.1, the range varying from 0 to 13 per subject. Multivariate linear regression analyses showed that the risk of OJ and OB reduction increased with nightly treatment time (Table 5). Furthermore, OJ reduction was positively related to female gender. The age- and gender-adjusted regression models explained 39% of the variation of OJ reduction and 37% of the variation of OB reduction. In the other multivariate regression analyses, the average nightly wear of OA was replaced by the frequent use of OA, but in this analysis, no significant associations were found.

**Table 3:** Changes in overjet and overbite after 1-year treatment of OAs in patients with obstructive sleep apnea (*n* = 40)

	Baseline mean (SD)	Follow-up mean (SD)	Difference mean (SD)	p*
Overjet, mm	2.7 (1.6)	2.4 (1.6)	− 0.4 (0.5)	< 0.001
Overbite, mm	3.3 (2.0)	3.1 (2.3)	− 0.3 (0.9)	0.070

^*^By paired *t*‐test

**Table 4:** Molar occlusion at baseline and in 1-year -follow-up in 40 patients with OSA

	Right side occlusion at baseline (*n*)	Right side occlusion at follow-up (*n*)	Left side occlusion at baseline (*n*)	Left side occlusion at follow-up (*n*)
Class I Class II	25 14	26 12	26 13	24 12
Class III Indefinable	0 1	1 1	0 1	1 3

**Table 5:** Predictive factors of dental changes after one-year of oral appliance therapy. Data are from multivariate linear regression model; the effects of age and gender were considered

	Overjet reduction			Overbite reduction		
	β	S.E.	*p*	β	S.E.	*p*
Gender, 0=female, 1=male	–0.637	0.151	0.010	–0.158	0.222	0.490
Age, years	–0.341	0.006	0.098	0.293	0.008	0.156
Average nightly wear of OA, hours	0.492	0.035	0.021	0.439	0.051	0.039
Mandibular protrusion with OA, mm	0.373	0.046	0.071	–0.285	0.067	0.166
	*R* ^*2*^=0.39			*R* ^*2*^=0.37		

*β* regression coefficient, *S.E.* standard error, *R*^2^ not adjusted

**Fig. 1:**
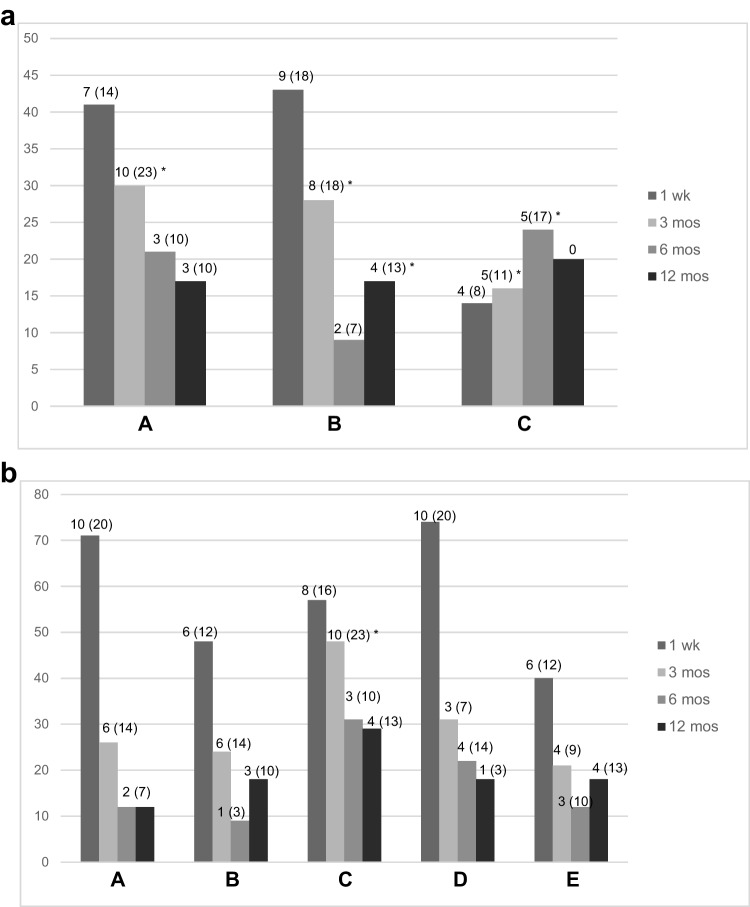
**a** Percentage of subjects who reported pain in TMJs (**A**), pain in muscles (**B**), and clicking (**C**) during OA therapy. The proportion of women *n* (%), and a significant difference in the prevalence between men and women (*) are announced in bar charts. **b** Percentage of subjects who reported pain in teeth (**A**), jaw stiffness (**B**), dry mouth (**C**), hypersalivation (**D**), and gingival irritation (**E**) during OA therapy. The proportion of women *n* (%) and a significant difference in the prevalence between men and women (*) are announced in bar charts

Almost half of the patients had TMD symptoms and discomfort of using OA during the first week of OA therapy (Fig. 1a and b). Hypersalivation, dry mouth, and pain in teeth were the most common side effects reported by the patients. After the first visit at 3-month follow-up, the frequency of most symptoms almost halved except in clicking, which showed to be a persistent TM disorder. Pain in TMJ and in muscles, clicking, and dry mouth were significantly more common among females than in males, especially at 3-month follow-up. Concerning the clinical TMD signs (Table 6), the frequency of muscle pain temporarily tripled within 3 months after starting OA therapy compared to the baseline, while in TMJ pain and in clicking, the number of patients with those signs remained about the same during the study. In the linear regression analyses, predictive factors for clinical TMD signs were younger age at 3-month follow-up, female gender, and larger mandibular advancement with OAs at 6-month follow-up and greater nightly OA adherence at 12-month follow-up (Table 7). The age- and gender-adjusted regression models explained 21% of TMD at the 3-month, 30% of TMD at the 6-month, and 38% of TMD at 12-month follow-up. In bivariate analyses, there was a moderate correlation between overjet and overbite reduction and average nightly use of OA (*r* = 0.4 and *r* = 0.5, respectively). At 6-month follow-up, clicking correlated positively (*r* = 0.39) to the frequent use of OA, hypersalivation correlated also positively (*r* = 0.63) to the average nightly use of OA, but muscle pain correlated negatively (*r* =  − 0.60 and *r* =  − 0.40) to frequent and nightly use of OA. At 12-month follow-up, there was a negative correlation between gingival irritation and frequent and nightly use of OA (*r* =  − 0.52 and *r* =  − 0.59).

**Table 6:** Clinical signs of TMD in obstructive sleep apnea patients using OAs in one-year follow-up

Signs of TMD	*n*	%
Pain in TMJ		
Baseline (*n* = 58)	2	3
3 months (*n* = 52)	5	10
6 months (*n* = 45)	3	7
12 months (*n* = 40)	1	3
Muscle pain		
Baseline (*n* = 58)	6	10
3 months (*n* = 52)	19	36
6 months (*n* = 45)	5	11
12 months (*n* = 40)	4	10
Clicking		
Baseline (*n* = 58)	16	28
3 months (*n* = 52)	11	21
6 months (*n* = 45)	12	27
12 months (*n* = 40)	14	35

**Table 7:** Predictive factors for TMD signs at the 3-month (model 1), 6-month (model 2), and 12-month follow-up (model 3) in obstructive sleep apnea patients using OAs

	Model 1 *β*	*p*	Model 2 *β*	*p*	Model 3 *β*	*p*
Gender, 0 = female, 1 = male	− 0.070	0.751	− 0.476	0.019	-0.167	0.470
Age, years	− 0.437	0.047	− 0.127	0.518	0.316	0.131
Mandibular protrusion with OA, mm	0.134	0.515	0.400	0.041	0.077	0.705
Average nightly wear of OA, hours	0.230	0.297	0.117	0.537	0.490	0.024
	*R*^2^ 0.21		*R*^2^ 0.30		*R*^2^* 0.38*	

*β* regression coefficient, *R*^2^ not adjusted

Data are from multivariate linear regression model adjusted by gender and age

## Discussion

This is the first study to evaluate if changes in occlusion in patients with OSA are related to objective adherence to OA use. Earlier review studies have found that the most reported OA side effects are dental, the risk of developing new TMD signs and symptoms with OA therapy is low, and pre-existing signs and symptoms of TMD do not seem to exacerbate with OA use [10, 19]. The OAs with 60% of mandibular advancement used in our study had a favorable effect on mild-to-moderate OSA [16], and there seemed to be only a minor effect on occlusion after 1‐year of OA therapy. The present study showed that reductions in OJ and OB, as well as clinical TMD, are significantly associated with a greater nightly adherence to OA but not with the frequent use of OA. To assess the wear time of OAs for clinical optimal treatment outcome without detrimental dental changes or exacerbation of TM-disorders in the future, objective measures of OA adherence together with investigating personal dental features influencing dental changes are needed.

### Effect of OA adherence on side effects

In OA therapy, there is no consensus describing adequate adherence. Based on self-reports, patients with OSA usually wear OAs at least 5 h a day and at least 4 days per week [11]. Although currently debated for CPAP-therapy, the treatment threshold of less than 4 h a day on less than 5 days a week is defined as non-adherence [20]. Our previous results showed that the objective wear time of OA (percentage use, mean (SD)) during the 1-year follow-up was 64% (37) [21]. The wear time of OA (hours/night, mean (SD)) in women was 6.2 (2.6) h, and among men 7.6 (1.1) h. The parameter that was positively related to higher objective adherence was more pronounced disturbance of snoring, while in mandibular retrusion, bruxism, and daily smoking, the relationship was negative. Surprisingly, excessive daytime sleepiness, side effects, or patients’ sociodemographic parameters were not associated with OA adherence [21].

The most common dental changes related to the use of OAs are the reduction in OJ and OB. When a subject wears an OA with dental arch coverage, the mandible tends to return to its natural position, which applies palatal force to maxillary incisors and labial force to mandibular incisors. Thus, dental changes are due to altered inclination of the incisors rather than skeletal changes or mandibular rotation [6]. This OA mechanism of action on the dentition is like that of functional orthodontic appliance used in growing subjects with Class II malocclusion. To achieve occlusal change, the target wearing time for the device is 12–14 h a day, and for minor dental effect, the wear of any device is 4 to 6 h per day [22]. As Cohen-Levy and co-workers found, a small force of 1.18 N per millimeter of advancement in OAs can induce dental movements [23]. Our earlier results showed that in the present sample, the objective nightly adherence of OA increased from 6.7 h at 3-month follow-up to 7.1 h at a 1-year study interval [21], which exceed the treatment duration threshold for dental changes. Earlier studies agree that there are progressive dental changes with prolonged OAs use [1, 6, 7]. The present results are parallel to earlier reports that especially the risk of OJ reduction is associated with OA treatment time but disagree the finding that dental changes are related to the frequent use of OA [1, 24]. This disagreement may be due to the finding that in this study, only half of the patients were frequent OA users after 12 months of therapy [21].

Like in other studies [2, 4] in the present report, the frequency of short-term TM disorders during acclimatization to OA therapy was high. In our study, the most common symptoms were hypersalivation and pain in teeth, which prevalence halved by the 3-month study interval; meanwhile, the prevalence of clicking and dry mouth remained almost unchanged during the 1-year study period. Interestingly, there was a clear difference in the prevalence of subjective and clinical TMJ pain, which most likely reflects difficulties in patients to differ TMJ pain from muscle pain. In the present report, subjects were not inquired about the subjective TMD symptoms at the baseline, but the initial signs of TMD were clinically evaluated. Despite a temporary increase in the frequency of muscle pain after OA therapy, the risk of developing new TMD signs with the use of OA proved to be low. Nevertheless, the results showed that the better the nightly adherence the higher the risk for clinical TMD signs. More specifically, a study on TM disorders in OSA patients suggests that TM dysfunction following OA wearing is related to altered muscle dynamics rather than changes due to increased stress in the temporomandibular joint itself [25]. Since early adherence to OSA treatment is a strong predictor of long-term adherence [26], an early check-up for identification and resolution of problems using OA is important.

To summarize, adherence to OA therapy is of utmost importance to ensure successful treatment for OSA. Generally, subjects who are less adherent are less susceptible to dental changes, but if the wear of OA is minor, OSA symptoms are likely to continue. At present, it is impossible to assess the target wearing time of OA to ensure the success of OA therapy and to avoid side effects.

### Effect of mandibular advancement on side effects

Earlier studies disagree of the proportional improvement of apnea–hypopnea index (AHI) by an increase of mandibular advancement [15, 27]. Our earlier results showed that AHI normalized in 51% of the patients with moderate mandibular advancement (60%), indicating effective treatment. The risk of OJ [22, 28], and OB reduction [5] is associated with the amount of mandibular advancement in the device, and side effects appear with over 50% of mandibular protrusion [15]. The present results showed that with 60% of mandibular advancement, OJ reduced about 0.4 mm in 1‐year study period, and the reduction in OB was even less (0.25 mm). In the earlier studies, the mean reductions of OJ and OB after 6 months of OA therapy was 0.87 mm and 0.61 mm [8], while the reductions in OJ and OB after several years of OA therapy varied from 0.7 to 1.22 mm and from 0.60 to 1.25 mm, respectively [6]. Regarding dental changes by OA therapy, also the number of occlusal contacts in (pre)molar region is likely to reduce, and the molar/cuspid occlusion tends to shift towards a more mesial occlusion [5]. In the present sample, the molar occlusion changed in only eight subjects, partly due to molar extraction, and in the regression analyses, mandibular protrusion with OA was not related to dental changes. To conclude, the present results show that by using SomnoDent Flex device with 60% of mandibular advancement, dental side effects are minor in 1‐year study period.

Device design may also influence the risk of occlusal changes. A flexible device without incisor coverage is likely to increase the irregularity of the lower incisors compared with a rigid OA with incisor coverage [29]. Marklund recommends a soft elastomeric device with a small vertical opening of the mandible to prevent large reductions in OJ and OB [1]. In the present study, all the patients used the SomnoDent Flex device, which is an acrylic device with soft inner liner, which is comfortable to teeth. This device allows free jaw opening but no lateral movements, a quality that appeals patients who are likely to be distressed of fixed appliances (i.e., monoblocks), but may be inconvenient for patients with bruxism. Regarding the degree of mandibular vertical opening in OAs, it has been found that larger (14 mm) incisal opening increases patients´ inconvenience and decreases device acceptance compared to 4 mm incisal opening [30]. In SomnoDent Flex, the vertical incisal opening is about 4 mm. Initially, all the patients had 60% of mandibular advancement of the OAs. After the second ambulatory polygraphic recodings with OAs in situ (after 3-month follow-up), the advancement was increased 1 mm in four patients and 2 mm in one patient, but no new TM disorders developed in these patients after the titration. As recently concluded, there is not one superior custom-made OA design in OSA treatment regarding AHI reduction, improvement in daytime sleepiness, adherence, patient preference, or side effects [31]. However, custom-made OA designs proved to be superior to thermoplastic OA designs because of higher rates of objective improvement and cure of OSA [32] and higher hours per night adherence [33].

Regarding signs and symptoms of TM disorders, the earlier Finnish study [12] showed that the subject’s TMD symptoms remained unchanged during 2-year follow-up with the mandibular advancement of 50% of maximum (mean protrusion was 5.4 mm). The present results are in line with those findings since the prevalence of subjective symptoms remained almost unchanged between 6- and 12-month follow-up with the mandibular advancement of 60% (mean protrusion was 5.8 mm). At 6-month follow-up, the larger mandibular protrusion increased the risk of clinical TMD signs.

### Effects of gender and age on side effects

The linear regression model showed that the risk of OJ reduction with OA treatment increased in women, which refers to significantly (*p* = 0.041) larger baseline OJ in women (3.5 mm) compared to that of men (2.5 mm). Although dental changes during OA therapy are more likely among elderly people due to age changes in their periodontal health [28], in the present study, age was not a risk factor of dental changes. In adult population, the prevalence of orofacial pain varies from 10 to 15% being twice as high in women compared to men [34, 35]. Gender difference in TMD prevalence may be due to genetic factors affecting pain vulnerability as well as hormonal and psychosocial determinants [35]. Since female patients tend to have more often TM disorders, they are also more prone to interrupt OA treatment than males [36]. In the present study at 3-month check-up, women had more often TMJ pain, muscle pain, and clicking than men, and in the linear regression analysis, they were more likely to have clinical TMD signs. During this study, four patients dropped out because of TM disorders; three of them were women. In our original study group, the age range varied from 26 to 73 years. The present finding that younger age (model 1) predisposes to TMD signs is parallel to the finding of Häggman-Henrikson and co-workers [35] that TM disorders are most prevalent among 35‐ to 50-year-olds.

### Effects of dentofacial features on side effects

The extent of dental movements with OA therapy is somehow related to the dentofacial features [1, 3, 9]. Marklund [1] found that patients with favorable occlusal bites are likely to develop OJ reduction with OA use. Therefore, she recommends for patients with normal occlusion a soft elastomeric device with an advancement less than 6 mm and low-to-moderate vertical displacement to diminish the risks of dental side effects. The present finding of larger baseline OJ in women increases the risk of OJ reduction partly agrees with the finding of Marklund, but in this study, the correlation between baseline OB and OJ/OB reduction was weak. Furthermore, it is suggested that in patients with fewer teeth and reduced periodontal health, dental changes are larger [9, 22], but in bivariate analyses of this study, the correlations between OJ and OB reduction and the number of missing teeth were weak (*r* =  − 0.06 and *r* =  − 0.07, respectively). The evaluation of the periodontal health was not included in the present study protocol.

## Conclusion

The present results showed that although OJ reduced significantly during the study period, the dental changes were minor with SomnoDent Flex device set at 60% of mandibular advancement. Further, OJ and OB reductions and clinical TMD signs were associated with a greater nightly adherence to OA therapy but not with the frequent use of OAs. To assess the wear time of OAs for clinical optimal treatment outcome without detrimental dental changes or exacerbation of TM disorders in the future, objective measures of OA adherence together with investigating personal dental features influencing dental changes are needed.

### Limitations of the study

This study has limitations. The power analyses of the original study showed that in the main outcomes (AHI reduction at least 50% and OA adherence at least 60%), with standard parameters of 80% power and *α* 0.05, 31 patients were needed. Considering the expected dropouts, the required sample size was estimated to be 40 patients. Concerning patients with available adherence data, attrition was larger than expected; therefore, the sample is small for this part of the study. Thus, the risk of bias of the results could not be eliminated. The follow-up time was short to conduct an investigation of the long-term dental side effects in patients using OAs. Unfortunately, not all the patients returned the questionnaires regarding subjective symptoms of TMD.

## Data Availability

Due to the nature of this research, participants of this study did not agree for their data to be shared publicly, so supporting data are not available.
